# Butein-instigated miR-186-5p-dependent modulation of TWIST1 affects resistance to cisplatin and bioenergetics of Malignant Pleural Mesothelioma cells

**DOI:** 10.20517/cdr.2022.56

**Published:** 2022-07-03

**Authors:** Mario Cioce, Daniela Rutigliano, Annamaria Puglielli, Vito Michele Fazio

**Affiliations:** ^1^Department of Medicine, Laboratory of Molecular Medicine and Biotechnology, University Campus Bio-Medico of Rome, Rome 00128, Italy.; ^2^Institute of Translational Pharmacology, National Research Council of Italy (CNR), Rome 00133, Italy.; **^#^** These authors contributed equally.

**Keywords:** Mesothelioma, butein, miR-186-5p, TWIST1, pithelial-to-mesenchymal transition (EMT), chemoresistance, cancer metabolism, invasion, anchorage-independent growth

## Abstract

**Aim:** Malignant pleural mesothelioma is a chemoresistant tumor, and biphasic and sarcomatoid histologies portend the worst prognosis for malignant pleural mesothelioma (MPM) patients. We obtained the microRNA expression profile of three biphasic-sarcomatoid MPM cell lines to identify commonly expressed microRNAs and evaluate the effect of butein, a chemo-sensitizing compound, on this microRNA subset.

**Methods:** Nanostring-based microRNA profiling and analysis through the ROSALIND platform were employed to identify the commonly modulated microRNAs and their targets. MicroRNA-mimic transfection, Luciferase assay, and Western blotting were employed to show specific perturbation of TWIST1 levels by miR-186-5p. Sphere-forming assays, invasion assay, and metabolic profiling were used to assess the biological consequences of the butein-instigated miR-186-5p-mediated perturbation of TWIST1 levels. TGCA analysis was used to search for the correlation between TWIST1 and miR-186-5p levels in biphasic and epithelioid MPM specimens.

**Results: **We identified a set of perturbed microRNAs, common to three biphasic/sarcomatoid MPM cell lines, after butein treatment. When focusing on miR-186-5p, we unraveled a butein-ignited and miR-186-5p-mediated modulation of TWIST1 levels which affected the 3D anchorage-independent growth, cisplatin resistance, invasion, and bioenergetics of the MPM cell lines tested. We showed that miR-186-5p and TWIST1 levels are anti-correlated in biphasic MPM specimens from TCGA.

**Conclusion:** We unraveled a novel mechanism of action of butein, which attenuated the pro-tumorigenic features of MPM at least through a miR-186-5p-TWIST1 axis. We suggest that those activities converge into the chemo-sensitizing effect of this compound and may be of translational relevance.

## INTRODUCTION

Mesothelioma is a neoplastic disease arising from the mesothelial linings of the pleural and peritoneal space. Its pathogenesis and progression envisage genomics alterations and environment-derived chronic inflammation in a complex interplay, with much left to be understood^[1,2]^. Three main histological presentations characterize malignant pleural mesothelioma (MPM), named epithelioid, biphasic, and sarcomatoid^[3]^. Rather than being separate entities, these histotypes may represent a structural–functional continuum where epithelial-to-mesenchymal transition (EMT) plays an important role^[4]^. EMT may correlate with specific pro-tumorigenic features^[5]^, which contribute to the worse prognosis of biphasic and sarcomatoid MPMs. In fact, the expression of EMT genes has prognostic significance in MPM^[6]^. MPM exhibits long clinical latency and significant resistance to therapy, with the latter impacting only to a limited extent the natural history of the disease. As a result, prognosis for MPM patients results in poor PFS and OS^[1,7,8]^. The mainstay of MPM treatment is still represented, for first-line or inoperable patients, by cisplatin and antifolate^[1,9]^. Ex vivo studies have shown that MPM cells are endowed with high resistance to therapy^[10]^; therefore, attenuating such a process is an unmet need. We and others showed that specific rearrangement of cell subpopulations, sustained by the acquisition of a senescence-associated secretory phenotype (SASP), may underlie the emergence of MPM chemoresistance^[11-14]^. We found that a naturally occurring compound with pleiotropic functions, butein (20,40,3,4-tetrahydroxychalcone), interfered with the emergence of those chemoresistant aldehyde dehydrogenase-positive (ALDH^pos^) cell subpopulations, by simultaneously blocking NFkB and STAT3 signaling^[15,16]^. Such interference with the intra-tumor heterogeneity of MPM translates into reduced adaptive stress responses and, ultimately, chemo-sensitizing effects, partially sustained by changes in the gene expression profile of the treated cells^[17]^. Butein is also known to exert chemo-sensitizing effects via AKT signaling and modulate the MAPK pathway through its antioxidant functions^[18-20]^. We also recently showed that butein modulates the levels of DNA repair genes, which further contributes to its chemo-sensitizing activity^[17]^. 

miRNAs are non-protein-coding single-stranded RNAs^[21]^. miRNAs bind generally to the 3’ untranslated region (3’-UTR) of mRNAs of target genes, thereby functioning as the negative posttranscriptional regulators of gene expression^[22]^. miRNAs modulate key processes of tumor initiation and progression, ranging from the acquisition of pro-metastatic features to metabolic reprogramming and chemoresistance^[23-26]^.

miR-186-5p is a debated miRNA since its role ranges from oncogenic to a tumor suppressive one, in a cancer-tissue and stage-specific manner^[27]^. There is an established link between miR-186-5p expression and resistance to therapy including cisplatin, taxol, and methotrexate^[28-33]^. miR-186-5p was shown to target twist-related-protein-1 (TWIST1), a key EMT-related transcription factor, in three different settings^[28,30,34]^. Changes in TWIST1 level could portend a poor prognosis in TCGA cohorts of several cancer settings^[6]^* .*TWIST1 modulates aerobic glycolysis in pancreatic cancer cells by increasing the expression of key glycolytic genes, including *HK2* and *PKM2*^[35]^. Insightfully, the ability of TWIST1 to impinge on EMT and metabolic reprogramming may converge toward the acquisition of chemoresistant phenotype. In fact, EMT is a key process toward the acquisition of chemoresistance^[36,37]^. TWIST1 is upregulated in MPM tumors and cell lines and may play a role in the development of MPM^[6,38]^. Further, vaccines against TWIST1 were recently shown to enhance CTLA-4 blockade in experimental mesothelioma immunotherapy approaches^[39]^.

Butein was shown by others and us to be capable of reversing chemoresistance to cisplatin and pemetrexed in MPM^[15,16,18]^. We hypothesized that butein exerted its chemo-sensitizing effects at least partially through microRNA modulation. Thus, we performed a microRNA expression analysis of three MPM cell lines with sarcomatoid and biphasic originating histotypes and identified 33 microRNAs modulated by butein. Among the identified targets, we focused on the miR-186-5p axis and demonstrated a miR-186-5p-mediated modulation of TWIST1 expression level. We showed that such modulation may explain at least in part the chemo-sensitizing effect of butein, thereby perturbing 3D anchorage-independent growth, cisplatin resistance, invasion, and bioenergetics of the MPM cells.

## METHODS

### Reagents

Butein (C_15_H_12_O_5_) was purchased from Cayman Chemicals (Ann Harbor, MI, USA) and dissolved in DMSO (Sigma-Aldrich, St. Louis, MO, USA). Cisplatin (CDDP) was purchased from Selleckchem (Houston, TX, USA).

### Cell culture

Cells were cultured in Ham’s F12 supplemented with L-glutamine 10% fetal calf serum (Gibco BRL, Grand Island, NY), 100 U/mL penicillin, and 100 µg/mL streptomycin in a humidified atmosphere containing 5% CO_2_ at 37 °C. The human MPM cell lines MSTO-211H, NCI-H2373, and HP1 were described previously^[12]^. All cell lines were in-house tested for mycoplasma contamination by using a commercially available PCR-based assay (R&D Systems, Minneapolis, USA).

### Cell metabolism

A Seahorse Bioscience XF24 Extracellular Flux Analyzer was used to measure the extracellular acidification rate (ECAR) and oxygen consumption rate (OCR). Logarithmically growing mesothelioma cell lines were maintained in normal complete growth media and seeded onto a gelatin-coated 24-well XF Flux Analyzer assay plate at 80,000 cells/well (NCI-H2373) or 40,000 cells/well (HP1 and MSTO.211H) 24 h prior to assay. These seeding numbers were determined based on the doubling time of the mentioned cell lines. Cells were switched to serum-free XF assay media (Seahorse Biosciences, Billerica, MA, USA) with 25 mM glucose, 1 mM sodium pyruvate, and 2 mM glutamine and placed in a CO_2_-free incubator at least 2 h before the assay. Multiple measurements were obtained at baseline and following injection, sequentially of glucose (10 mM), oligomycin (2 μM ), and 2-deoxyglucose (100 mM) (Merck Life Science, Milan, Italy) for ECAR measurement and oligomycin (1 μM), FCCP (0.25 μM), and rotenone (1 μM) (Merck Life Science, Milan, Italy) for OCR measurement. Values are reported as mpH/min for ECAR and pmoles/min for OCR.

### Western blotting assay

Whole cell extracts (40-50 µg) from cells or tissues were separated by SDS-PAGE and then transferred to polyvinylidene difluoride membranes (PVDF; Bio-Rad, Hercules, CA, USA). The membranes were blocked and then probed with antibodies against TWIST1 (ab50887) and anti-alpha tubulin (ab176560) as a loading control (Abcam, Cambridge, MA, USA). After washing, the blots were incubated with horseradish peroxidase-conjugated secondary antibodies.

### Transfection of mimic-186-5p

The miR-186 mimics (mirVana® miRNA mimic) and its negative control (mirVana™ miRNA Mimic Negative Control) (ctrl) mimics were from ThermoFisher (Waltham, MA, USA). Cells were seeded in 60 mm dishes and transfected (25 nM each) in Ham’s F12 with reduced serum [2% fetal bovine serum (FBS)] using the JetPrime reagent (Polyplus Transfection, New York, NY) before being processed for downstream analyses.

### Luciferase 3-UTR assays

The TWIST1 3’ UTR fragment sequence containing the binding site with miR-186-5p was cloned into the pMIR-reporter vector (Addgene, Cambridge, MA). A quick-change site-directed mutagenesis kit (Stratagene, CA, USA) was used to mutate the miR-186-5p binding site. For reporter assays, the MPM cell lines and the HEK293 cells (as a technical control for higher transfection efficiency) were transiently co-transfected with the TWIST1 3’ UTR luciferase vector or mutant 3’ UTR with miR-186 mimic or ctrl by JetPrime reagent (Polyplus Transfection, New York, NY). The firefly luciferase activities were measured consecutively using the Luciferase Reporter assay system (Promega, Madison, NJ, USA), according to the manufacturer’s protocol. The percentage of luc activity in the cells transfected with miR-186-5p mimics over the cells transfected with the ctrl mimics was reported.

### Invasion assay

Cell invasion was assessed using a Matrigel invasion assay. Briefly, diluted Matrigel matrix was carefully added to the center of each Transwell® insert (8 μm PET membrane, Corning, NY, USA) for invasion assays. Cells were starved of serum for 24 h and then seeded in triplicate. Lower chambers contained serum-free medium or medium supplemented with 20% FBS. The inserts were washed twice with PBS1X before fixing and staining in crystal violet solution for 15 min and then air-dried. The invaded and migrated cells were observed and imaged under a microscope. The bound crystal violet was eluted with 33% acetic acid and the eluent absorbance at 590 nm was measured. 

### Sphere-forming assay

For generating cell spheroids, a variable number of single cells/well were seeded into BIOFLOAT TM 96-well plates (FaCellitate, Germany) in DMEM-F12/1:1 + Glutamax supplemented with B27 (no RA), BSA, bFGF (20 ng/mL), and hEGF (10 ng/mL) (Life Technologies Inc., Grand Island, NY, USA).

### Assessing chemo-sensitivity to cisplatin

For the determination of IC_50_, formed MPM spheroids at Passage 2 were incubated in media with or without the addition of cisplatin (0-100 µM) for 12 h before drug withdrawal. After 72 h, the IC_50_ was defined as the cisplatin dose capable of reducing by 50% the average number of spheroids from four to six independent 96 wells.

### Viability assay

Cells were shortly pulsed with butein (10 µM) or ctrl (0.01% DMSO) for 8 h and viability was assessed by flow cytometry-based detection of Sytox-Blue positive cells (Sytox Blue Dead cell stain, Thermo Fisher, CA USA) at 12, 24, 48, 72, and 96 h after drug withdrawal.

### RNA expression analysis

Analysis was performed on all samples using the nCounter Analysis System (NanoString Technologies, Seattle, WA, USA) and the nCounter Human v2 miRNA Panel that contains 798 unique miRNA barcodes. Probes for housekeeping genes such as ribosomal protein L10 (RPL10), beta-actin (ACTB), beta-2-microglobulin (B2M), glyceraldehyde 3-phosphate dehydrogenase (GAPDH), and ribosomal protein L19 (RPL19) were used as internal controls.

### ROSALIND® nanostring miRNA expression analysis

Data were analyzed by ROSALIND® (https://rosalind.bio/), with a HyperScale architecture developed by ROSALIND, Inc. (San Diego, CA). Normalization, fold changes, and *P*-values were calculated using criteria provided by Nanostring (Seattle, WA, USA). Following background subtraction based on POS_A probe correction factors, normalization was performed in two steps: positive control normalization and codeset normalization. During both steps, the geometric mean of each probeset was used to create a normalization factor. ROSALIND calculated fold changes and *P*-values for comparisons using the *t*-test method. *P*-value adjustment was performed using the Benjamini-Hochberg method of estimating false discovery rates (FDR).

### Statistical analysis

Where appropriate, statistical analysis was performed using Student’s *t*-test and *P* ≤ 0.05 were considered statistically significant. Group analysis was performed by ANOVA and Prism GraphPad software.

### Principal component analysis

For creating principal component analysis (PCA) plots, Clustvis was employed https://biit.cs.ut.ee/clustvis.

## RESULTS

### Butein treatment modulated microRNA expression levels

**Figure 1:**
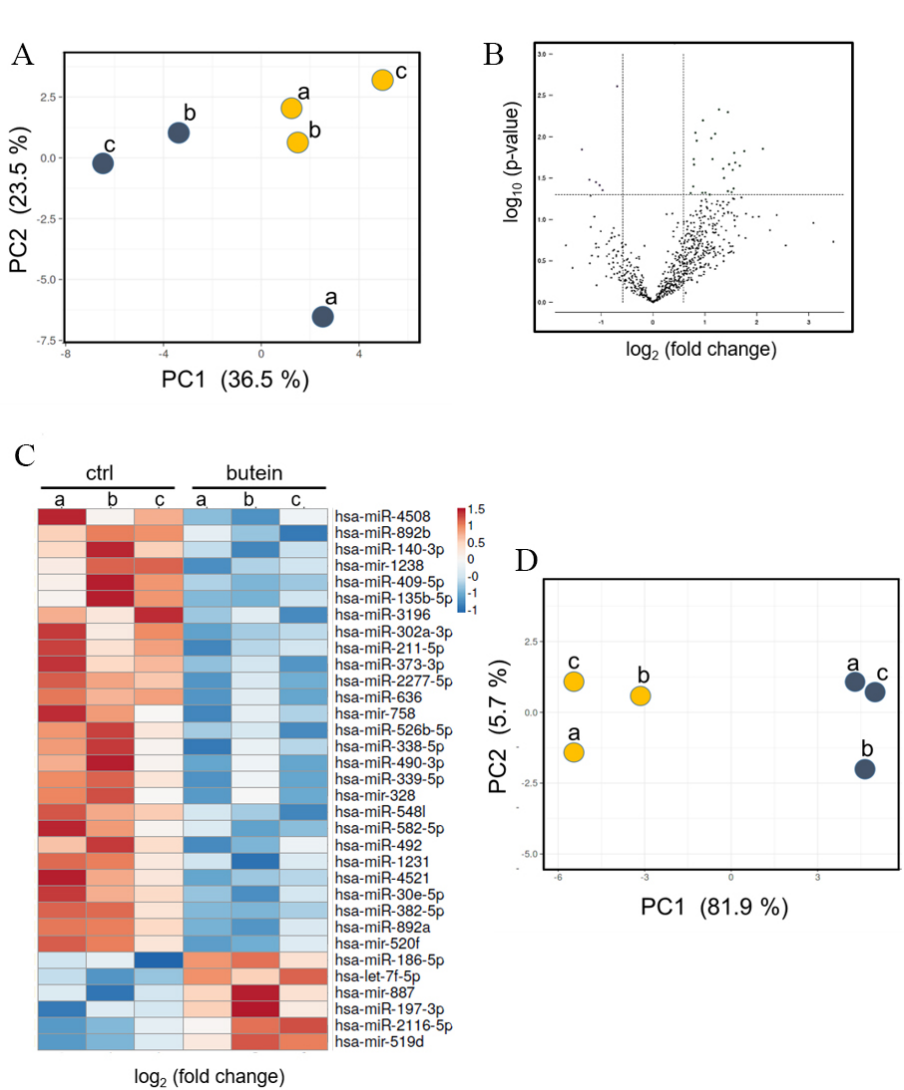
Butein treatment modulated microRNA expression levels. (A) PCA plot of the microRNAs expressed in: HP1 (a); NCI-H2373 (b); and MSTO-211H (c). Blue indicates samples treated with ctrl (DMSO 0.01%), while yellow indicates samples treated with butein for 8 h at 10 µM. (B) Volcano Plot showing the microRNAs commonly modulated in all three MPM cell lines and whose perturbation by butein reached statistical significance. (C) Heatmap showing the normalized levels of the microRNAs common to the three MPM cell lines, treated as in (A). Log2 fold changes are reported. The average of the two experiments is shown. (D) PCA plot showing the distribution of the three MPM cell lines treated as in (A), after selecting the commonly modulated miRNAs.

We performed microRNA profiling of three MPM cell lines: NCI-H2373, HP1, and MSTO-211H [Figure 1]. The three cell lines shared a biphasic (HP1, MSTO211H) and sarcomatoid (NCI-H2373) originating histo-type, both associated with worse prognosis and lower response to therapy. The three MPM cell lines exhibited different microRNA expression patterns, as assessed by PCA [Figure 1A]. We interrogated this microRNA expression profile to identify a set of microRNAs common to all three cell lines, thereby hypothesizing that commonly modulated sets of microRNAs can predict functions common to the biphasic/sarcomatoid cyto-type. We thus identified a limited set of commonly expressed microRNA (*n* = 33, Figure 1B and C and Supplementary Table 1). Butein treatment, executed at non-apoptotic concentrations and schedule of administration^[17]^ [Supplementary Figure 1A], determined a clear perturbation of microRNA levels, as assessed by the PCA analysis [Figure 1B and C]. This revealed a clear separation, on the main PC component (PC1: 81.9%), of the three MPM cell lines as a function of butein treatment [Figure 1D]. When focusing on microRNAs significantly modulated by butein treatment, we observed that microRNA downregulation prevailed after treatment with butein, with a much smaller subset (*n* = 6) of microRNAs upregulated by butein [Figure 1C]. 

### TWIST1 is a miR-186-5p target in butein-treated MPM cells

**Figure 2:**
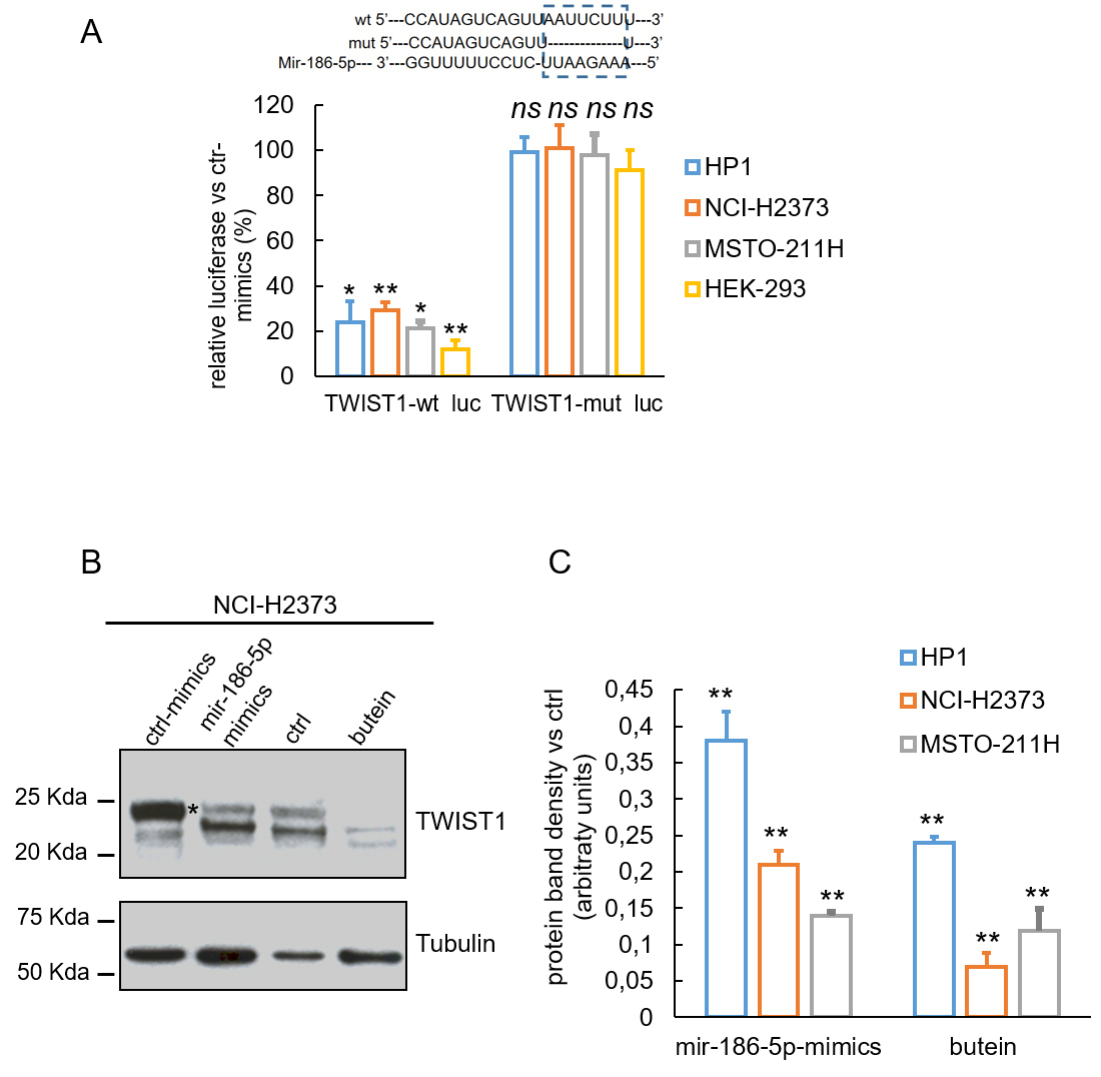
TWIST1 is a miR-186-5p target. Inset: The putative binding site of miR-186-5p within the Twist1 3’-UTR is shown, with the paired sequences of the wt and mutant (mut) constructs generated. (A) The pMIR-Twist1-WT and pMIR-Twist1-MT vectors were transfected into the three MPM cell lines and in HEK293 cells along with miR-186-5p or ctrl mimics and luciferase activity was assessed 48 h later. The percentage of luciferase activity in the cells transfected with miR-186-5p mimics over the cells transfected with the ctrl-mimics is reported. Means ± SD of three replicates are shown. *P < 0.05, **P < 0.01. (B) Twist1 protein levels in NCI-H2373 cells treated with ctrl or miR-186-5p mimics or butein and ctrl (DMSO 0.01 %) as assessed by Western blotting. Anti-tubulin staining was used as a loading control. Asterisk (*) indicates the TWIST1-specific protein band. (C) Histograms reporting the relative (normalized) protein band intensity of TWIST1 from all three MPM cell lines treated as indicated in (B). The average values of two independent experiments are reported. **P < 0.01 (*vs*. ctrl).

Among the upregulated microRNAs, we focused on miR-186-5p for its important contribution to EMT and chemoresistance in other cancer settings and because one of its targets, TWIST1, is an important factor in MPM progression^[40,41]^ and a target of butein^[16]^. To verify that TWIST1 was a target of miR-186-5p, we co-transfected the three MPM cell lines and a non-MPM cell line (HEK293, as a control) with either miR-186-5p mimics or its control (ctrl mimics) [Supplementary Figure 1B] at 25 nM and with expression vectors containing luciferase under the control of the TWIST1 3’-UTR region, wild-type (wt), or mutated (mut) [Figure 2A, inset]. The luciferase activity in the MPM cells transfected with miR-186-5p mimics was significantly lower than that in the cells with control sequences [Figure 2A]. HEK293 cells exhibited the highest degree of luciferase downregulation, possibly as a consequence of increased transfection efficiency [Figure 2A]. To confirm that the reduced luciferase activity was caused by miR-186-5p binding to the seed site of 3’-UTR, the seed sequence of TWIST1 3’-UTR was mutated in the luciferase reporter construct. No significant changes in the luciferase activity were recorded when the mutated 3’-UTR-luc construct was co-transfected with the miR-186-5p mimics [Figure 2A]. We performed Western blotting experiments in NCI-H2373 cells transfected with either ctrl or miR-186-5p mimics or treated with ctrl (DMSO) or butein. We found that both butein treatment (10 µM for 8 h) and miR-186-5p mimics similarly affected the TWIST1 protein levels, when compared to their respective controls [Figure 2B], and this held true for all three MPM cell lines tested [Figure 2C].

### Butein-instigated increase of miR-186-5p affected the sphere forming ability and the cisplatin sensitivity of 3D-grown MPM cells

**Figure 3:**
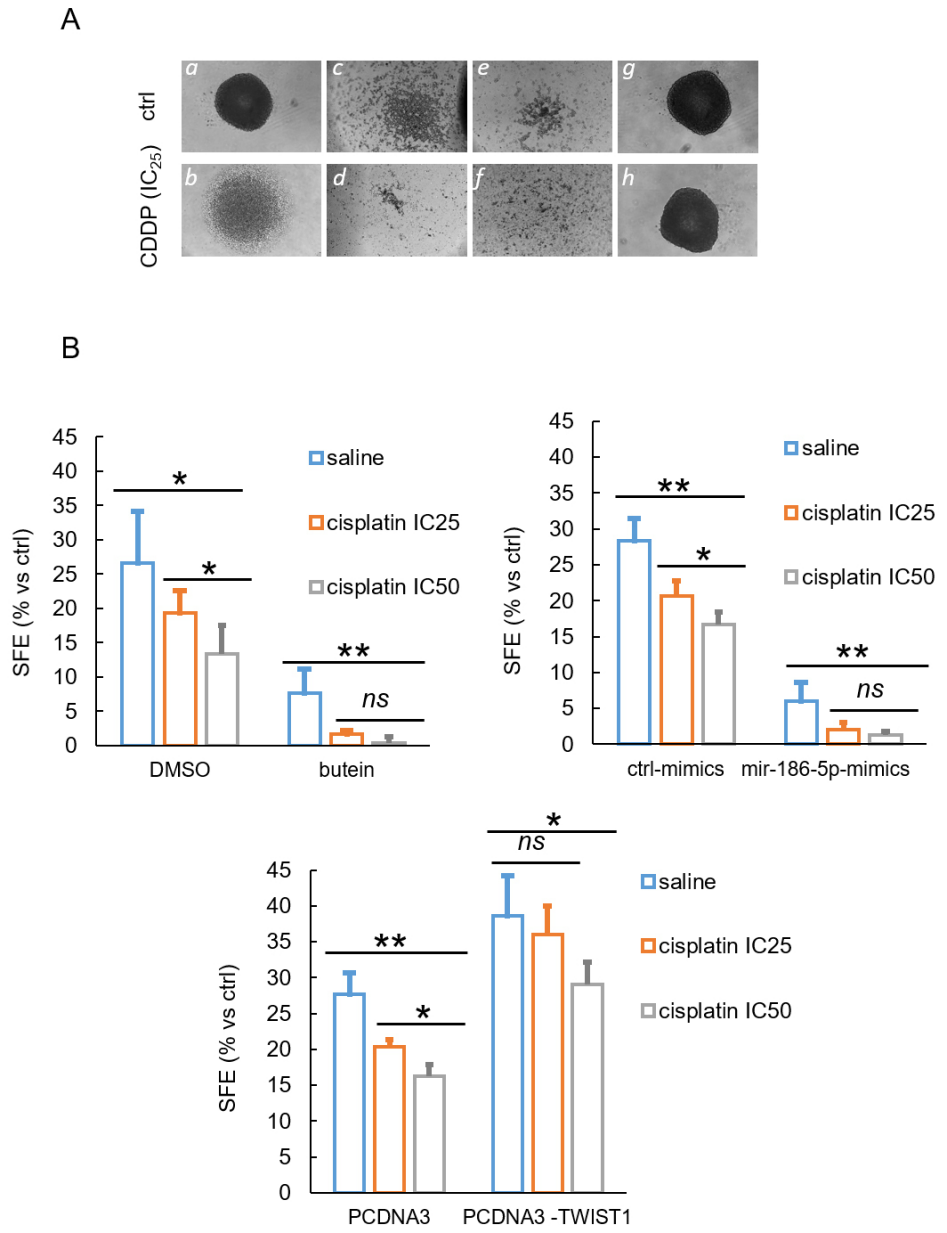
Butein-instigated increase of miR-186-5p affected the sphere forming ability and the cisplatin sensitivity of 3D-grown NCI-H2373 cells. (A) Spheroid formation assay: NCI-H2373 cells, treated with ctrl-(DMSO 0.01%) (*a, b*) or butein (10 µM, 8 h) (*c, d*), transfected with miR-186-5p mimic (*e, f*), or with PCDNA3-TWIST1(*g, h*), were clonally seeded and allowed to form spheroids for 48 h. After that, ctrl (saline: *a, c, e, g*) or cisplatin (*b, d, f, h*) was added at the IC_25_. Representative micrographs of the formed spheroids (on Day 2 after cisplatin or saline treatment). Scale bar, 200 µm. (B) Histograms showing quantitation of the SFE from NCI-H2373 cells treated with saline or cisplatin at IC_25_ and IC_50_, respectively, and counted on Day 7 after treatment started. The percentage is relative to the control sample within the group except when otherwise indicated. Asterisk indicate statistical significance as follows: *P < 0.05; **P < 0.01; ns: not significant (*P* > 0.05). The average of 3 experiments is shown.

To study the effect of butein-instigated miR-186-5p-dependent TWIST1 modulation on pro-tumorigenic MPM features, we evaluated the effect of the mentioned treatments on the resistance of MPM 3D spheroids to cisplatin. Spheroid cultures are enriched for the expression of EMT factors and may represent a suitable system to study chemoresistance^[12,42,43]^. MPM cells were grown as 3D spheroids after seeding in anchorage-independent, quasi-clonal densities, and sphere forming efficiency (SFE) was evaluated for saline- and cisplatin-treated spheroids after a 12 h pulse of the drug at IC_25 _and IC_50 _doses (empirically determined for each cell line, Supplementary Figure 2A) [Figure 3]. Evaluation of the size, appearance, and number of the formed spheroids after an additional 48 h revealed a clear change in the morphology of the cisplatin- *vs*. ctrl-treated spheroids (Figure 3A, top and bottom, respectively), with the cisplatin treated ones being much less compact and rounded and significantly reduced in number [Figure 3A]. Butein treatment (10 µM, 8 h) [Supplementary Figure 2B] induced spheroid disaggregation in the ctrl-treated cultures, which was even more dramatic in the samples co-treated with cisplatin, suggesting an effect on cisplatin sensitivity of the spheroids [Figure 3A and B]. Transfection of the miR-186-5p constructs induced very similar effects when compared to butein, consisting of spheroid disaggregation in ctrl-treated samples, which was more prominent in the cisplatin treated ones [Figure 3A and B]. On the other hand, overexpression of TWIST1 induced the formation of round and compact spheroids, which were significantly resistant to cisplatin treatment, as evaluated by morphology and number [Figure 3A and B]. We observed similar effects when challenging the spheroids obtained from MSTO-211H and HP-1 MPM cell lines with a similar treatment scheme [Supplementary Figure 3A and B]. Thus, butein could both attenuate the SFE and reduce the resistance of MPM spheroids to cisplatin, at least partially through a miR-186-5p-mediated downregulation of TWIST1.

### TWIST1 is a critical effector of miR-186-5p-mediated inhibition of invasion

**Figure 4:**
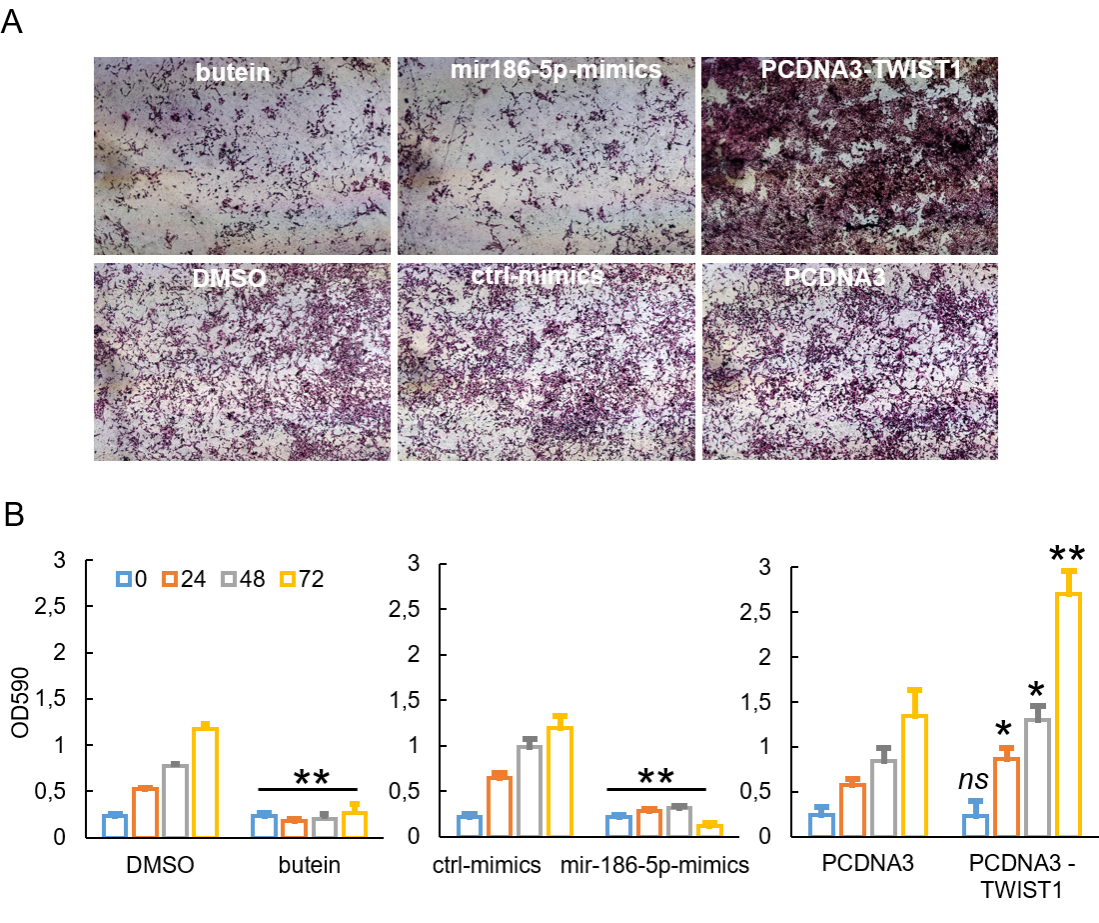
Butein-instigated miR-186-5p-mediated inhibition of TWIST1 attenuated invasion of NCI-H2373 cells.** (**A) Representative bright-field images of Transwell invasion assay inserts 48 h after seeding of the NCI-H2373 cells. Cells were stained with crystal violet. (B) Histograms showing quantitation of the migrated NCI-H2373 cells treated as in (A) and counted at 24, 48, and 72 h after treatment started. The bound crystal violet was eluted and the absorbance at 590 nm was measured using a plate reader. The average of three experiments is shown. The percentage is relative to the relative control group except where otherwise indicated. Asterisk indicate statistical significance as follows: *P < 0.05; **P < 0.01; ns: not significant (*P* > 0.05).

Next, we evaluated the effect of butein-instigated miR-186-5p-dependent TWIST1 modulation on the invasive properties of NCI-H2373 cells [Figure 4]. Staining of the cells which invaded the Matrigel-coated surface and evaluation of the optical density in time revealed that, when compared to ctrl (DMSO), butein strongly affected NCI-2373 invasion [Figure 4A], and this inhibition appeared as early as 24 h after cell treatment and lasted over time [Figure 4B]. Overexpression of TWIST1 strongly increased the invasive ability of the NCI-H2373 cells, as compared to the cells transfected with the vector alone [Figure 4A], and this was maximal at 72 h after the transfection [Figure 4B]. These results were consistent in MSTO-211H [Supplementary Figure 4], while the HP1 cells did not show relevant invasion at steady state and were not tested further (data not shown). Thus, butein could inhibit the invasion of MPM cells at least partially through a miR-186-5p-mediated downregulation of TWIST1.

### A butein-instigated increase of miR-186-5p modulated oxidative mitochondrial respiration and glycolytic activity of MPM cells

**Figure 5:**
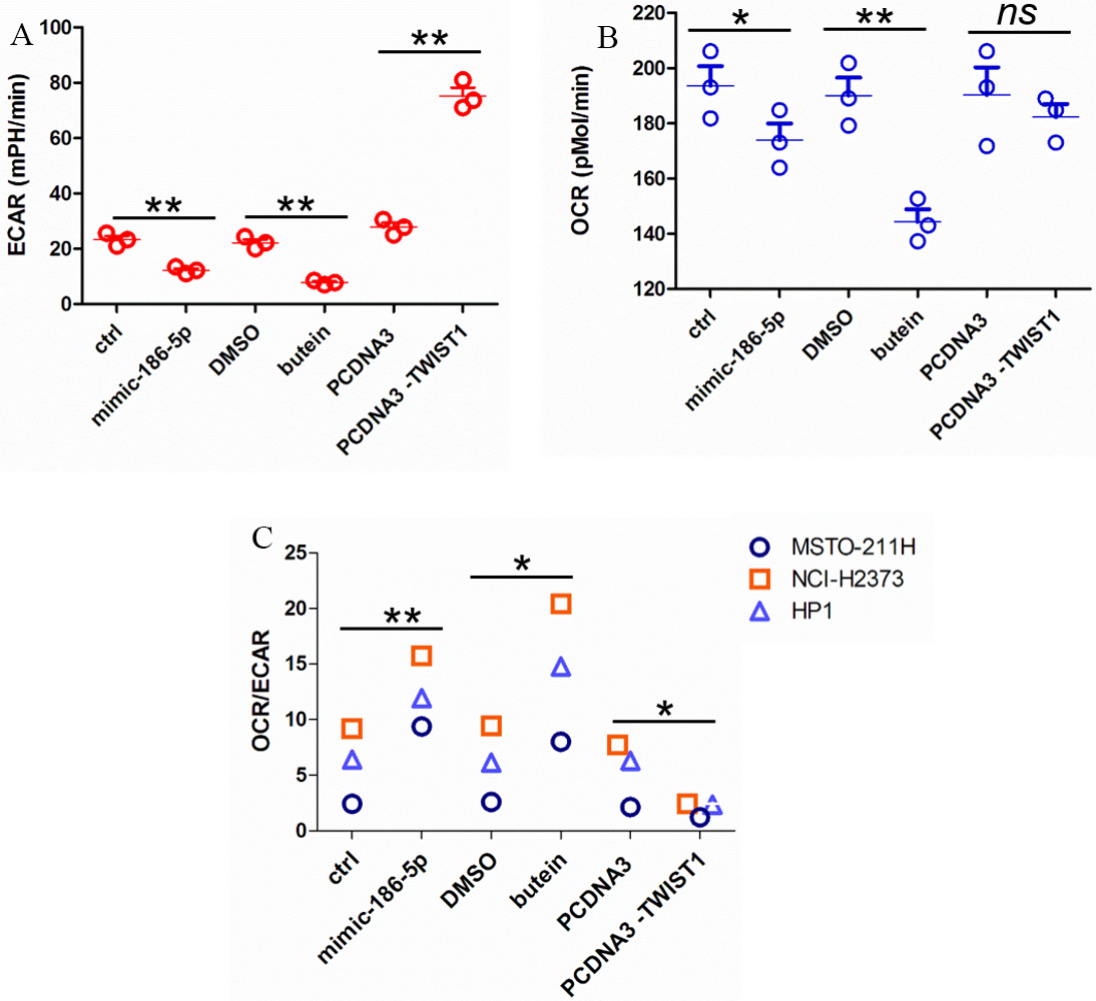
A butein-instigated increase of miR-186-5p modulated oxidative mitochondrial respiration and glycolytic activity of MPM cells. (A, B) Basal ECAR and OCR measured in NCI-H2373 cells treated or transfected as indicated. Values are reported as mpH/min for ECAR and pmoles/min for OCR Results are shown as the mean ± SEM of three independent experiments. Asterisk indicate statistical significance as follows: *P < 0.05, **P < 0.01). (C) OCR to ECAR ratio assessed for all three MPM cell lines of this study.

Next, we evaluated the effect of butein, miR-186-5p, and TWIST1 on the bioenergetics of the NCI-H2373 cells. We evaluated the basal ECAR and OCR of NCI-H2373 cells treated with ctrl or butein and transfected with ctrl or miR-186-5p mimics or TWIST1 [Figure 5A and B]. We found that butein treatment and, similarly, miR-186-5p mimics reduced the glycolytic flux of the NCI-H2373 cells [Figure 5A]. Both butein and miR-186-5p mimics reduced the OCR of NCI-H2373 [Figure 5B]. However, butein treatment affected the OCR rate to a larger extent than did the miR-186-5p mimics, suggesting that the deeper OCR inhibition by butein may involve additional mechanisms. TWIST1 overexpression did not significantly affect the OCR of NCI-H2373 cells (*P* = 0.07) while readily increasing their glycolytic flux [Figure 5A and B].

We extended our observations to the additional MPM cell lines MSTO-211H and HP1 [Supplementary Figure 5A and B]. This revealed that all three cell lines exhibited a significantly different metabolic profile, with NCI-H2373 being more reliant on mitochondrial oxidative respiration and MSTO-211H being more glycolytic. HP1 showed an intermediate metabolic profile [Supplementary Figure 5A and B]. Despite those differences, evaluation of the basal OCR and ECAR revealed that, in MSTO-211H and HP1 cells as well, butein and miR-186-5p mimic acted very similarly by reducing the glycolytic flux, with TWIST1 enhancing the latter in an opposite way. The OCR was affected to a larger extent by butein than by miR-186-5p mimics [Supplementary Figure 5C and D]. Overexpression of TWIST1 significantly reduced the OCR in MSTO-211H and HP1 cells (*P* < 0.05), possibly as a consequence of the increased glycolytic flux following TWIST1 overexpression [Supplementary Figure 5D]. Those changes were evident by evaluating the OCR/ECAR ratio for all three cell lines [Figure 5C].

Altogether, we found that butein treatment and the transfection of miR-186-5p mimics exerted very similar effects on the 3D anchorage-independent growth, cisplatin resistance, invasion, and bioenergetics metabolism of the three MPM cell lines tested. Conversely, overexpression of TWIST1 induced rather opposite effects by enhancing all the mentioned pro-tumorigenic properties.

### miR-186-5p and TWIST1 exhibited opposite behavior in biphasic MPM specimens

**Figure 6:**
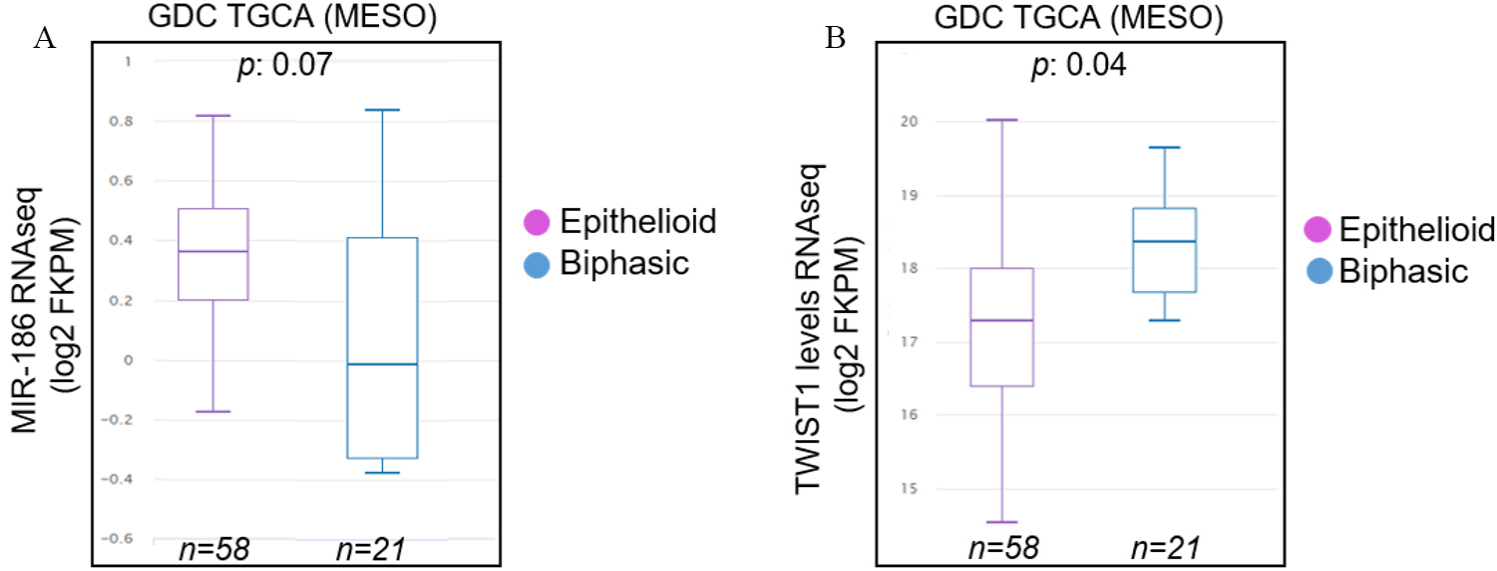
miR-186-5p and TWIST1 anti-correlated in biphasic MPM specimens. (A) Box plot reporting the levels of miR-186-5p assessed by RNAseq and expressed as Log2 (FKPM) in biphasic (*n* = 21) *vs*. epithelioid (*n* = 58) MPM specimens. (B) Box plot reporting the levels of TWIST1 mRNA assessed and reported as in (A) from the same MPM specimens. *P*-values are reported above each graph.

Finally, we explored the connection between miR-186-5p and TWIST1 in a more clinically relevant setting. When searching for a correlation between miR-186-5p and TWIST1 mRNA levels in the TGCA database, no significant correlation was observed between miR-186-5p and TWIST1 mRNA levels in MPMs (Spearman rho = -0.1936, *P* = 0.0748). However, we found a trend, for biphasic mesotheliomas (*n* = 21) toward exhibiting lower miR-186-5p (0.05 < *P* < 0.10) and higher TWIST1 expression, as compared to epithelioid mesotheliomas (*n* = 58) [Figure 6A and B].

## DISCUSSION

We addressed the microRNA profile of three mesothelioma cell lines. We chose to profile biphasic/sarcomatoid cell lines because the originating histotype of those cell lines is linked to a worse prognosis and fewer data are available when compared to the epithelioid histotype MPMs^[44]^. We uncovered a set of microRNAs commonly and significantly modulated in the mentioned cell lines. We found miR-186-5p as significantly and highly modulated by butein treatment. This focused our attention, given the involvement of one of the miR-186-5p targets, TWIST1, on both the biology of MPM^[38,40]^ and the possibility of explaining the mechanisms behind the chemo-sensitizing effects of butein^[16]^. This helped us delineate a butein-instigated modulation of TWIST1 by miR-186-5p which impinges on the invasion, 3D growth, chemoresistance, and metabolic features of the MPM cells.

EMT is an important feature of MPM, since mesothelial cells show a proclivity to undergo EMT even in pathophysiological conditions, such as peritoneal dialysis^[45,46]^. We and others showed that EMT sustains and accompanies a therapy-induced SASP which fuels the resistance of MPM cells to cisplatin and pemetrexed^[11,14]^. More recently, an EMT-omic signature emerged as s distinct prognostic trait of MPM^[47]^ and as a determinant of anti-CTLA-4 immuno-response^[11,14,39]^. TWIST1, being a key EMT player and a target of microRNA modulation by butein, may well find a place within the chemo-sensitizing actions of this versatile compound. In fact, we showed that, besides the effect on cell invasion and 3D growth, butein-modulated TWIST1 affects cisplatin resistance. A similar effect has recently been shown in epithelial ovarian cancer cells, where TWIST1 delineates a chemoresistant ovarian cancer phenotype^[48-50]^.

In addition to the mentioned anticancer effects, we found that the butein-miR-186-5p axis modulated MPM cell bioenergetics, by reducing glycolytic processing and mitochondrial respiration in all 3 cell lines tested. The degree of metabolic perturbation was similar in all three cell lines but varied in magnitude, according to differences in the three cell lines, already at steady state [Supplementary Figure 5]. Our investigation was limited to assessing ECAR and OCR rates. However, we, by using a more comprehensive metabolic assessment, and others, in different experimental settings^[51]^, showed how specific lipid species may mediate chemoresistance of MPM cells by activating NFkB signaling^[12]^. Even if there was no demonstration that TWIST1 modulation may directly affect the release of signaling lipids, the fact that butein, TWIST1, and miR-186-5p are involved in chemoresistance and the high degree of connection between metabolic pathways suggests that this may be the case and prompts future detailed investigation.

Here, we identified a novel action of butein, which is the microRNA modulation. Such findings are in line with what we and others showed on the anticancer action of butein, i.e., that it inhibits migration, invasion, clonogenicity, and resistance of the cells to chemo-therapeutics^[18]^. The effect of butein on TWIST1 matches what is known of the ability of TWIST1 to impinge on AKT signaling and drive resistance to cisplatin^[50]^. However, this may not be the only mechanism for butein: for example, target prediction of the butein-modulated microRNAs suggested that additional targets may mediate the chemo-sensitization effect of butein (data not shown). In line with this, the effect of butein treatment on the OCR was similar but stronger when compared to that of the miR-186-5p mimic [Figure 5B]. Butein has pleiotropic, metabolic effects including modulation of lipid biosynthesis through NFkB/STAT3 inhibition and TGF-beta-PPARγ interference^[52]^ and HMOX1 induction^[53]^. Therefore, there is the possibility that butein affects OCR through miR-186-5p-independent mechanisms. Thus, engagement of additional microRNA-target modules and a broader metabolic action may also explain the more profound effects of butein on the OCR of the MPM cell lines when compared to miR-186-5p mimics alone [Figure 5B]. On the other hand, gene expression profiling has already shown how butein may modulate DNA damage associated and DNA repair pathways^[17]^, and it is very likely that several mechanisms converge onto the anticancer action of butein, possibly with different kinetics in time and with tumor-stage specificity *in vivo*.

An unsolved question within this work is how butein may modulate the levels of miR-186-5p. There is some indication that a transcriptional mechanism may be responsible for this. Resistin (RETN) is a proinflammatory cytokine secreted from adipocytes and monocytes^[54]^. In addition to its pivotal role in inflammation-related diseases, RETN was shown to suppress the miR-186-5p levels, thereby contributing to cancer resistance in ovarian cancer^[55]^ and facilitating VEGF-C-associated lymphangiogenesis in human chondrosarcoma cells^[56]^. RETN expression is downregulated by an NFkB-mediated transcriptional mechanism in human monocytes^[57]^. Relevant to this, induction of RETN after butein treatment was recently shown in 3T3 mouse preadipocytes^[53]^. It is possible that butein, as being a known NFkB inhibitor, modulates RETN levels, thereby attenuating the resistin-mediated downregulation of miR-186-5p.

One limitation of this study is that we did not investigate whether the modulation of miR-186-5p by butein takes place in specific cell subpopulations. In fact, we showed that butein exerts differential effects on FACS sorted, chemoresistant MPM cell subpopulations, such as the ALDH^bright ^MPM cells^[17]^. Another limit of this study is that we did not address mechanistically how TWIST1 downregulation by butein may affect the pro-tumorigenic program. Since TWIST1 functionally interacts with AKT, and we and others showed that AKT is a downstream collector of survival signaling in pemetrexed-treated MPM cells^[50,58]^, it is possible that a TWIST1-AKT axis may be effective even in this experimental setting.

Finally, we did not make use in this work of primary MPM specimens, which may represent a more clinically relevant experimental setting. However, knowledge in the literature exists that TWIST1 was significantly increased in a gradient fashion when analyzing epithelioid, biphasic, and sarcomatoid primary MPMs^[40]^. We expanded on these data by observing that TWIST1 mRNA levels are anti-correlated with miR-186-5p levels in TGCA biphasic but not in epithelioid MPM specimens [Figure 6A and B].

## DECLARATIONS

### Availability of data and materials

The datasets and certain material used and/or analyzed during the present study are available from the corresponding author on reasonable request.

### Copyright

© The Author(s) 2022.
