# Supplementary material for: Butein-instigated miR-186-5p-dependent modulation of TWIST1 affects resistance to cisplatin and bioenergetics of Malignant Pleural Mesothelioma cells
Source: Cancer Drug Resist. 2022 Jul 3;5(3):814–28. doi: 10.20517/cdr.2022.56 (PMC9511809; doi:10.20517/cdr.2022.56)
Supplement: Supplementary file 1 [file cdr-5-3-814-SupplementaryMaterials.pdf]

Suppl. figure 1

A

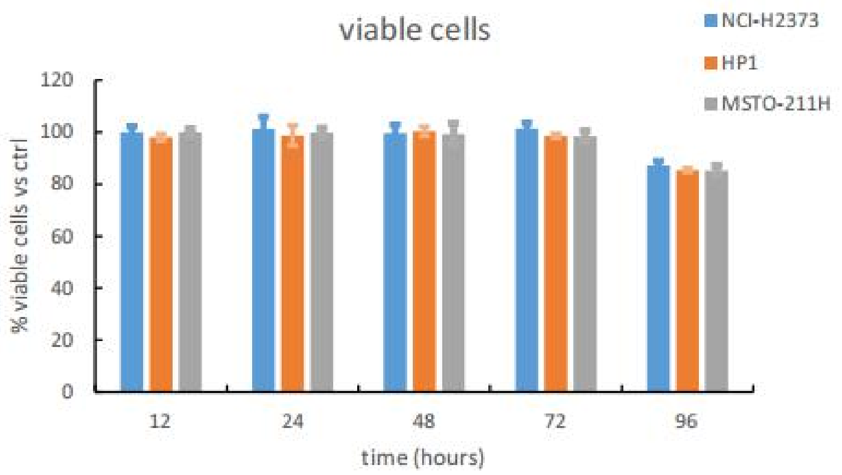

B

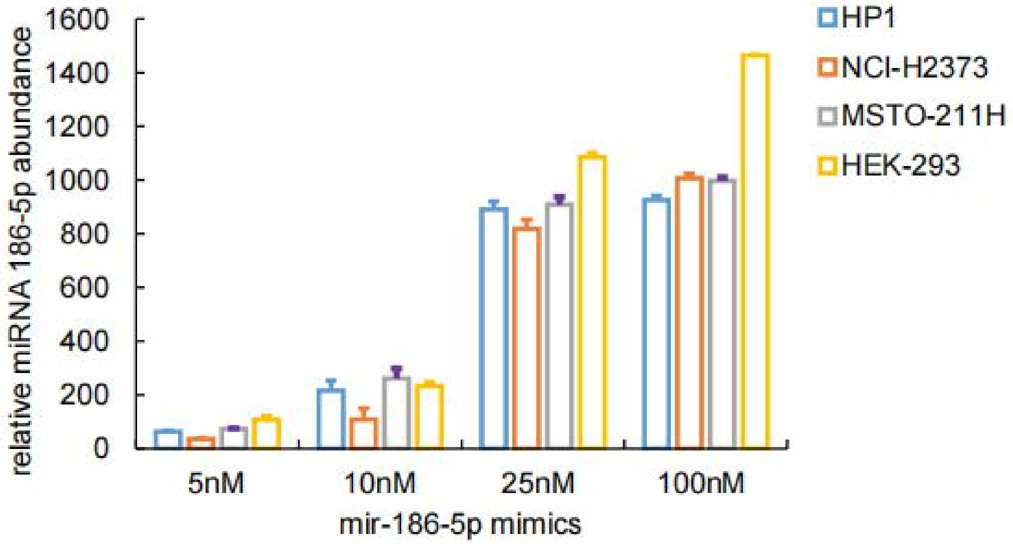

Figure S1. (A) Short-term treatment with butein does not affect the viability of MPM cells. Cells treated with butein (10 µM, 8 h) were assessed for viability at the indicated times after drug withdrawal by using a flow cytometry-based Sytox blue staining. (B) Levels of miR-186-5p reached after miR-186-5p mimic transfection at the indicated doses. Histograms reporting the relative miR-186-5p levels detected by QRT-PCR in HEK 293T, NCI-H2373, MSTO-211H, and HP1 cells transfected with

increasing amounts of miR-186-5p mimics. Relative levels of miR-186-5p- vs. ctrl-mimic transfected cells are reported. The average of three independent experiments is shown.

Suppl. figure 2

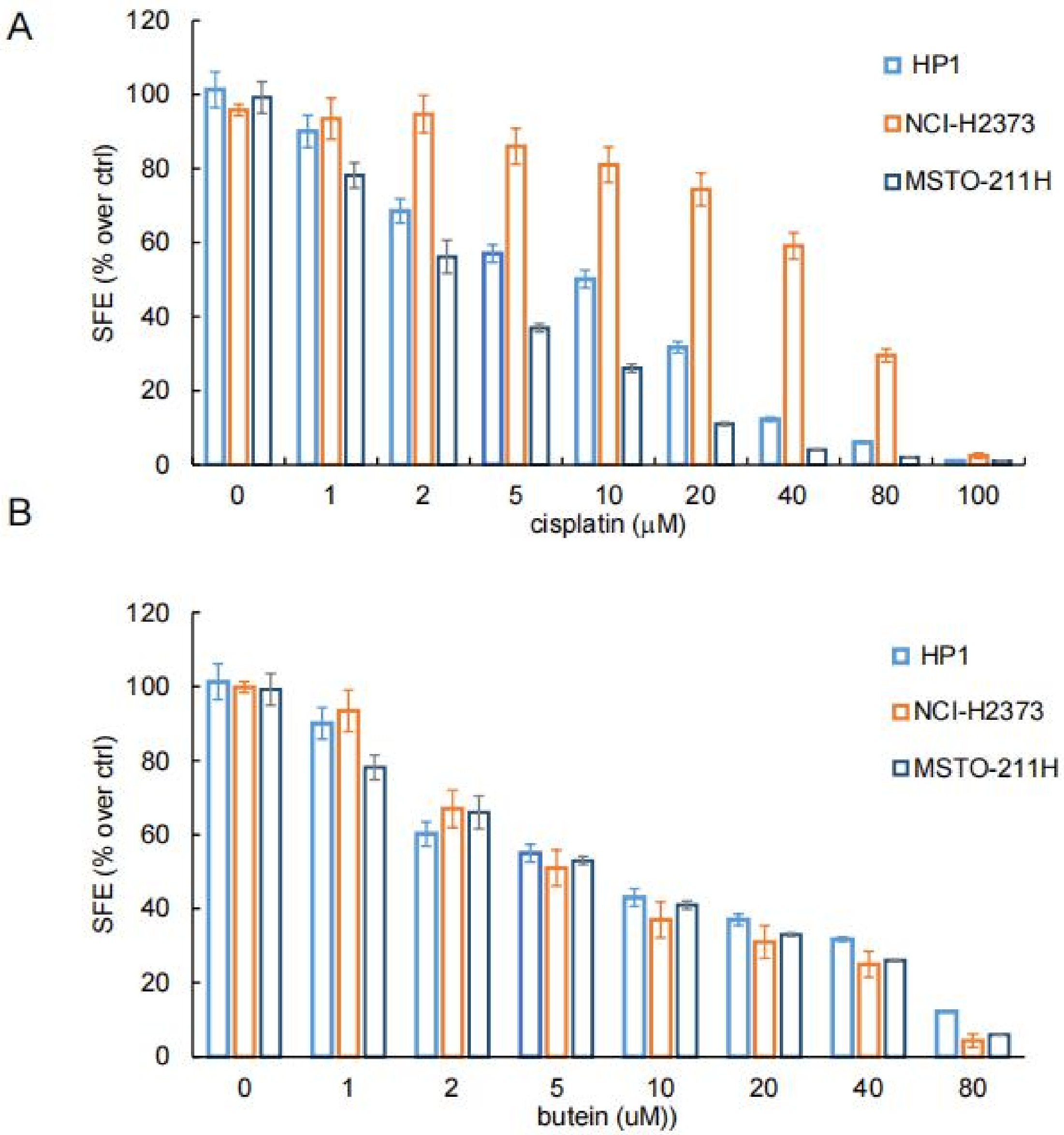

Figure S2. (A) Cisplatin treatment affected spheroid forming efficiency (SFE) of mesothelioma cells. Briefly, NCI-H2373, MSTO-211H, and HP1 cells were

quasi-clonally seeded in anchorage-independent conditions, and after 48 h, the formed spheroids were mechanically disaggregated and treated 24 h later with ctrl (saline) or cisplatin at the indicated concentration. The number of formed spheres from 4–6 independent wells is reported. Graphs show quantitation of the SFE from the treated MPM cells. Results are shown as the mean ± SEM of two independent experiments. The IC_{25}//IC_{50} for cisplatin was calculated for all three MPM cell lines and the results were 2.5/4.8 μM for MSTO-211H, 5/9.4 μM for HP1, and 25/53 μM for NCI-H2373, respectively. **(**B) Butein treatment affected SFE of mesothelioma cells. Briefly, NCI-H2373, MSTO-211H, and HP1 cells were grown as indicated in (A) and treated 24 h later with DMSO (0.05%) or butein at the indicated concentration. The number of formed spheres from 4–6 independent wells is reported. Graphs show quantitation of the SFE from the treated MPM cells. Results are shown as the mean ± SEM of three independent experiments.

Suppl. figure 3

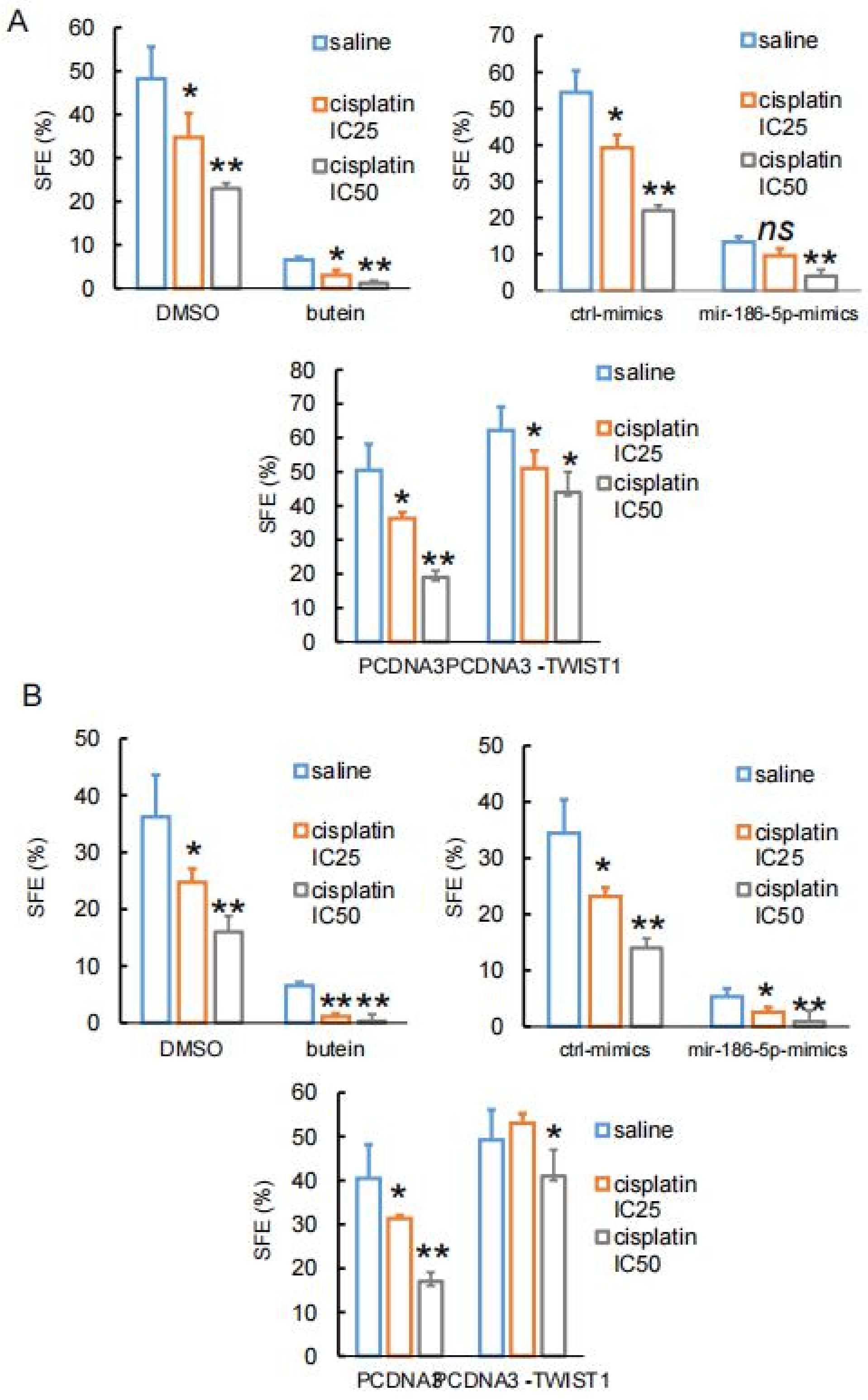

Figure S3. Butein-instigated increase of miR-186-5p affected the SFE and the cisplatin sensitivity of 3D-grown MPM cells. Histograms show quantitation of the SFE from HP1 (A) and MSTO-211H (B) cells treated as in Figure 3B and counted on

Day 7 after treatment started. * p < 0.05; ** p < 0.01; ns, not significant (p > 0.05). The average of three experiments is shown.

Suppl. figure 4

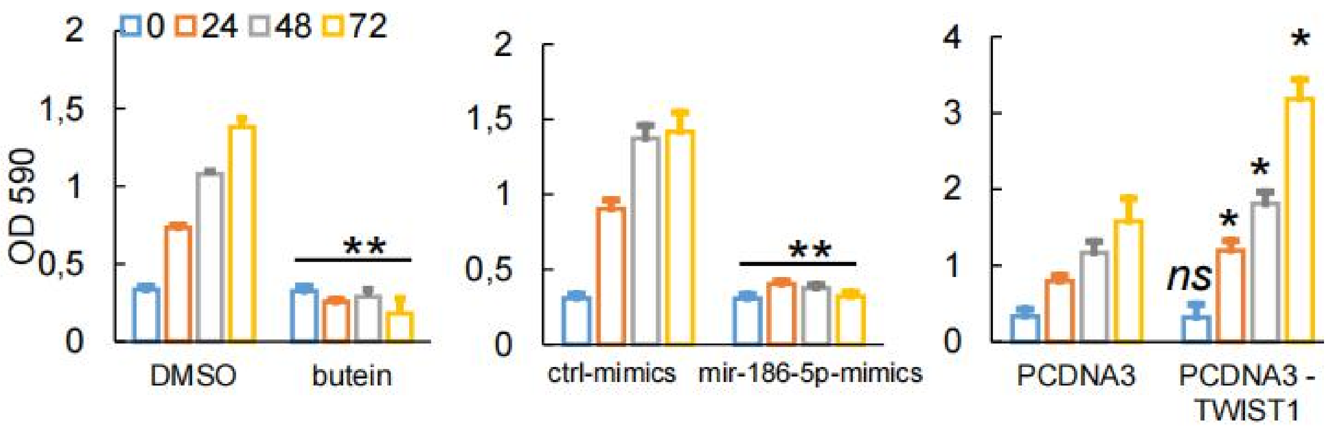

Figure S4. Butein-instigated miR-186-5p-mediated inhibition of TWIST1 attenuated invasion of MSTO-211H cells. Histograms show quantitation of the migrated MSTO-211H cells treated as in Figure 4B and counted at 24, 48, and 72 h after treatment started. The bound crystal violet was eluted and the absorbance at 590 nm was measured using a plate reader. * p < 0.05; ** p < 0.01; ns, not significant (p > 0.05). The average of three experiments is shown.

Suppl. figure. 5

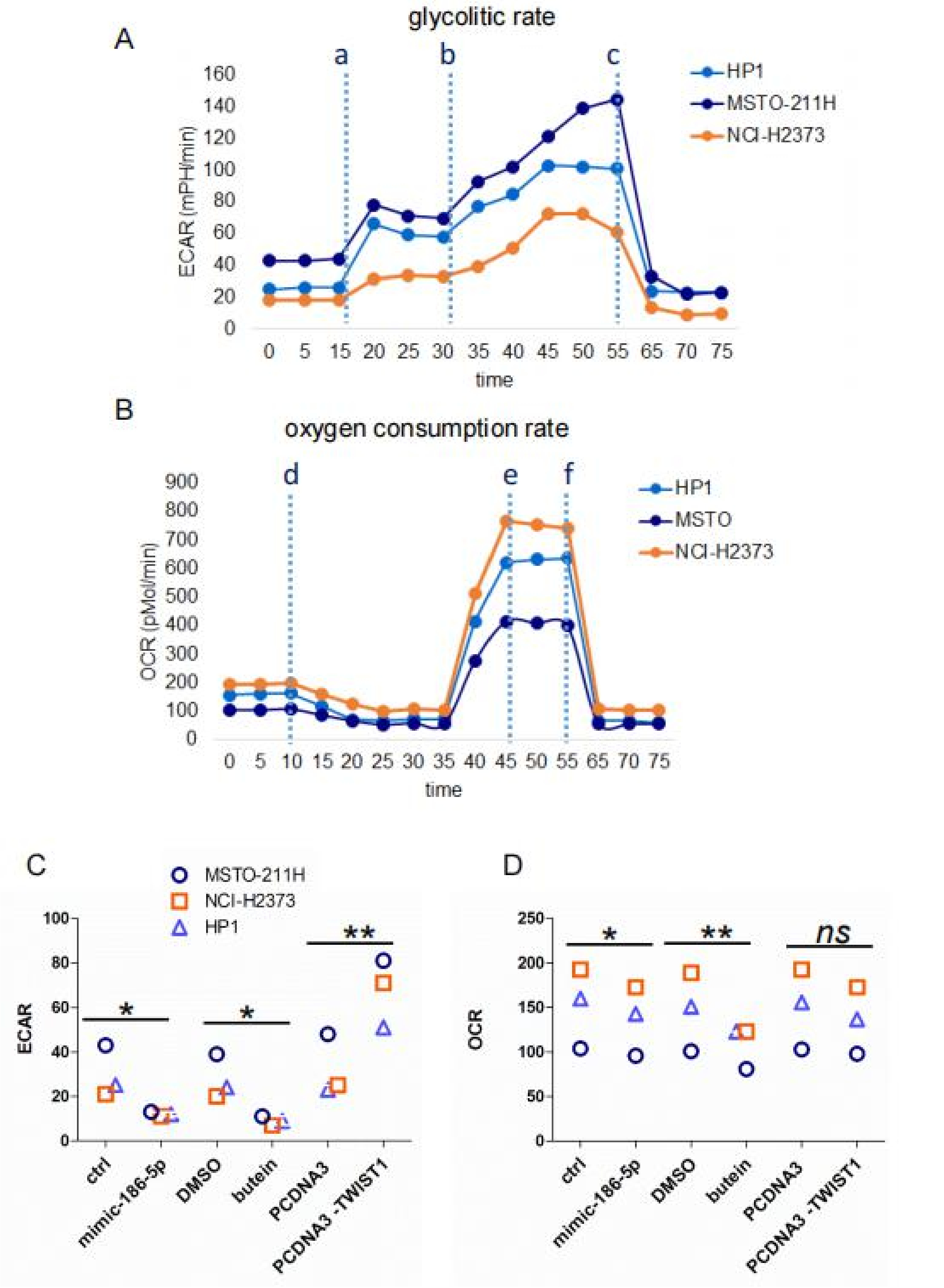

Figure S5. Butein-induced miR-186-5p-mediated TWIST1 modulation affected some metabolic features of MPM cells. (A) ECAR measured over time in the three MPM cell lines. Dotted lines indicate the addition of: glucose (10 mM) (a); oligomycin A (2 µM) (b); and 2-deoxyglucose (100 mM) (c) B. Oxygen consumption rate (OCR)

measured over time in the same MPM cell lines. Dotted lines indicate the addition of: oligomycin A (1 μM) (c); FCCP (0.25 μM) (d); and rotenone (1 μM) (e). Multiple measurements were obtained at baseline, and values reported as mpH/min for ECAR and pmoles/min for OCR. (C,D) ECAR and OCR measured in the three MPM cell lines treated or transfected as indicated. Values reported as mpH/min for ECAR and pmoles/min for OCR. Results are shown as the mean ± SEM of three independent experiments (* $p < 0.05$, ** $p < 0.01$). (C) OCR to ECAR ratio assessed for all three MPM cell lines of this study.

Gene Name	MSTO-211H-DMSO	HP1-DMSO	NCI-H2373-DMSO	MSTO-211H butein	HP1-butein	NCI-H2373-butein
hsa-miR-526b-5p	1.39718	0.27907	0.9608	-1.36694	-0.4733	-0.79681
hsa-miR-490-3p	1.387518333	0.047018333	0.728748333	-1.014031667	-0.120391667	-1.028861667
hsa-miR-339-5p	1.135704667	0.280624667	0.962354667	-1.043453333	-0.149815333	-1.185415333
hsa-miR-338-5p	1.368571667	0.054061667	0.905721667	-0.685058333	-0.206448333	-1.436848333
hsa-miR-2277-5p	0.676071667	0.499061667	0.988151667	-0.825018333	-0.346418333	-0.991848333
hsa-miR-636	0.871543	1.016463	1.283163	-1.307615	-0.413977	-1.449577
hsa-miR-492	1.237441667	0.382361667	0.578671667	-0.164108333	-0.855428333	-1.178938333
hsa-miR-140-3p	1.369988333	0.514908333	0.459678333	-0.546141667	-1.237461667	-0.560971667
hsa-miR-409-5p	0.97234	0.60268	0.06203	-0.52875	-0.63511	-0.47319
hsa-miR-4521	0.642836667	0.202796667	1.206456667	-0.536323333	-0.642683333	-0.873083333
hsa-miR-892a	0.76644	0.35194	0.78574	-0.31318	-0.867	-0.72394
hsa-miR-1231	1.090308333	0.235228333	1.179998333	-0.410781667	-1.517141667	-0.577611667
hsa-miR-4508	0.04235555	0.77223555	1.45397555	-0.13680445	-1.24316275	-0.88859945
hsa-miR-302a-3p	0.174943333	0.904823333	1.223993333	-0.589176667	-0.695536667	-1.019046667
hsa-miR-30e-5p	0.364563333	0.209923333	0.650643333	-0.229636667	-0.599026667	-0.396466667
hsa-miR-211-5p	0.244918333	0.559758333	0.878928333	-0.349281667	-0.455641667	-0.878681667
hsa-miR-3196	0.127318333	0.687278333	0.369008333	-0.636801667	-0.158201667	-0.388601667
hsa-miR-373-3p	0.338625	0.483545	0.972635	-0.840535	-0.361935	-0.592335
hsa-mir-328	0.84701	-0.00807	0.67367	-0.69471	-0.02347	-0.79443
hsa-mir-758	0.427916667	-0.012123333	0.669606667	-0.336203333	-0.120633333	-0.628563333
hsa-mir-520f	0.513191667	0.143531667	0.602881667	-0.180538333	-0.509298333	-0.569768333
hsa-miR-382-5p	0.512661667	0.143011667	0.519891667	-0.325458333	-0.431818333	-0.418288333
hsa-miR-548l	0.439103333	0.320993333	0.680793333	-0.740056667	-0.431376667	-0.269456667
hsa-miR-582-5p	0.423236667	0.027586667	0.709316667	-0.433993333	-0.540353333	-0.185793333
hsa-miR-892b	0.834248333	0.756778333	0.438518333	-1.152261667	-0.673661667	-0.203621667
hsa-mir-1238	0.804601667	0.797511667	0.116681667	-0.374558333	-0.480918333	-0.863318333
hsa-miR-135b-5p	0.855416667	0.540906667	0.029996667	-0.323743333	-0.567603333	-0.534973333
hsa-miR-186-5p	-0.171876667	-1.026956667	-0.345226667	0.233923333	0.712533333	0.597603333
hsa-let-7f-5p	-0.47478	-0.32986	-0.23309	0.46154	0.20933	0.36686
hsa-miR-197-3p	-0.226555	-0.316105	-0.914475	0.130335	0.973355	0.353445
hsa-miR-2116-5p	-0.647285	-0.180435	-0.820635	0.873995	0.767635	0.006725
hsa-mir-887	-1.131708333	-0.401828333	-0.305058333	0.274091667	1.167731667	0.396771667
hsa-mir-519d	-0.783263333	-0.316413333	-0.956613333	0.844936667	1.018676667	0.192676667

Suppl. Table 1. Listo of normalized levels of the commonly modulated microRNAs in the indicated MPM cell lines.

Table S1. Listo of normalized levels of the commonly modulated microRNAs in the indicated MPM cell lines
